# Trends in Premature Mortality Due to Alcoholic Cardiomyopathy Among Working Adults Aged 25-64 Years in the United States, 1999-2024: A CDC Wide-Ranging Online Data for Epidemiologic Research (WONDER) Analysis

**DOI:** 10.7759/cureus.117544

**Published:** 2026-10-06

**Authors:** Shivankur Singh, Sudesh Prajapathi, Mithilesh Yadaw, Aditi Mohta, Prashant Thandi, Shakti Singh, Manoj K Bhakhar, Bhushan Shah, Ashish K Jain, Akshyaya Pradhan

**Affiliations:** 1 Cardiology, All India Institute of Medical Sciences Bhopal, Bhopal, IND; 2 Cardiology, Dr. Ram Manohar Lohia Institute of Medical Sciences, Lucknow, IND; 3 Community Medicine, People's College of Medical Sciences and Research Centre, Bhopal, IND; 4 Cardiology, King George's Medical University, Lucknow, IND; 5 Cardiology, Leelawati Memorial Hospital, Bhopal, IND

**Keywords:** alcohol, alcoholic cardiomyopathy, cardiovascular disease, cdc wonder, epidemiology, mortality, premature mortality, united states

## Abstract

Background

Alcoholic cardiomyopathy (ACM) is an important alcohol-related cardiovascular condition associated with heart failure and premature mortality. However, long-term national trends in ACM mortality among working-age adults and changes during the COVID-19 pandemic remain incompletely characterized.

Methods

We conducted a retrospective population-based analysis of United States (US) mortality data from the Centers for Disease Control and Prevention Wide-ranging Online Data for Epidemiologic Research (CDC WONDER) database. Deaths with ACM (International Classification of Diseases, Tenth Revision [ICD-10] code I42.6) as the underlying cause of death were identified for 1999-2024. Adults aged 25-64 years were considered the working-age population. Crude mortality rates (CMRs) and age-adjusted mortality rates (AAMRs) were calculated per 1,000,000 population, with analyses stratified by sex, ethnicity (Hispanic origin), and age subgroup.

Results

Between 1999 and 2024, there were 13,741 deaths attributed to ACM, including 9,705 (70.63%) among adults aged 25-64 years. The pooled premature CMR was 2.3 per 1,000,000 population (95% CI: 2.2-2.3). Men accounted for 85.24% of premature deaths, with a pooled CMR of 3.9 per 1,000,000 compared with 0.7 per 1,000,000 among women. Adults aged 45-64 years accounted for 78.79% of premature deaths and had a CMR of 3.8 per 1,000,000 compared with 0.9 per 1,000,000 among those aged 25-44 years. Mortality increased during 2020-2021, reaching a peak in 2021, followed by a progressive decline through 2024. In 2024, the premature CMR was 1.9 per 1,000,000. AAMRs largely followed CMR trends.

Conclusion

ACM accounted for a substantial proportion of premature alcohol-related cardiovascular mortality in working-age adults in the US. Mortality was disproportionately concentrated among men and adults aged 45-64 years, increased during 2020-2021, and subsequently declined. These findings highlight persistent disparities and the need for continued prevention, early recognition, and targeted reduction of alcohol-related cardiovascular risk.

## Introduction

Alcoholic cardiomyopathy (ACM) is a recognized cardiovascular complication of chronic alcohol abuse and is relevant to the prevention of heart failure and alcohol-related cardiovascular mortality, with consequences extending beyond the immediate clinical outcome [1,2]. It is not limited to a diagnosis of reduced cardiac function; in a substantial proportion of patients, it also limits day-to-day functioning and lowers quality of life [3]. In adults still of working age, the associated disability and premature death also carry a measurable burden in lost work capacity and reduced productivity [4]. Specifically, deaths among adults aged 25-64 years represent premature loss during economically and socially active years, representing a broader burden to population health [5].

Assessing the burden and trends of ACM mortality may help identify the population groups in which earlier recognition and alcohol-related risk reduction are most relevant, particularly given the concentration of deaths in working-age adults. Greater clinical attention to ACM in these groups, including closer surveillance and earlier intervention in high-risk individuals, could reduce premature cardiovascular mortality.

This study aimed to assess the burden and trends in premature mortality due to ACM among adults aged 25-64 years in the United States (US) between 1999 and 2024, with subgroup analyses by sex, origin (ethnicity), and age groups, and patterns observed during the pre-pandemic, pandemic-era, and post-pandemic periods.

## Materials and methods

This retrospective, population-based study used the Centers for Disease Control and Prevention Wide-ranging Online Data for Epidemiologic Research (CDC WONDER) database [6,7]. CDC WONDER provides national mortality and population data derived from US death certificates, with one underlying cause of death recorded for each death. Data were extracted in July 2026 from the publicly available underlying cause of death mortality files. Since this study involved secondary analysis of de-identified aggregate-level primary data, institutional ethics approval was not required.

In CDC WONDER query settings, the index condition was ACM recorded as the underlying cause of death in the US between 1999 and 2024, identified using the International Classification of Diseases, Tenth Revision (ICD-10) code I42.6. The study population comprised adults aged 25-64 years, representing the adult working-age population. Deaths in this age group were considered premature adult mortality. All-age mortality data were also extracted to calculate the annual proportion of ACM deaths occurring among adults aged 25-64 years.

The variables extracted in Microsoft Excel included year of death, number of deaths, population denominators, crude mortality rate (CMR), age-adjusted mortality rate (AAMR), sex, ethnicity (Hispanic origin), and age subgroup. Sex was categorized as male or female. Ethnicity (Hispanic origin) was grouped as “Hispanic or Latino” or “Non-Hispanic or Latino” in this analysis. CDC WONDER had an additional category, “not stated.” Across the 26 study years, death counts for the not stated category were suppressed in 23 years, while zero deaths were reported in 2006, 2012, and 2024. CDC WONDER reported the corresponding population denominator as “not applicable”; therefore, neither an exact aggregate count of deaths with Hispanic origin not stated nor the proportion of the population represented by this category was calculated from the public output. The Hispanic-or-Latino mortality rate for 2000 was additionally designated “unreliable” by CDC WONDER and was treated as unavailable for annual rate interpretation. Suppressed cells were not reconstructed or back-calculated. Records in which Hispanic origin was not stated were excluded from the Hispanic origin-specific analysis. Overall, sex-specific and age-specific analyses were obtained independently without restricting records according to Hispanic-origin status; thus, these records were not intentionally excluded from those analyses. Race was not included as a primary stratification because exploratory CDC WONDER extraction demonstrated substantial suppression or statistical unreliability for several racial categories owing to small annual ACM death counts.

Age-specific comparisons were made between adults aged 25-44 years and those aged 45-64 years. Mortality rates were reported per 1,000,000 population. Pooled CMRs were calculated by dividing the total number of deaths by the sum of the annual population denominators over the study period and multiplying by a million. The annual premature mortality proportion was calculated by dividing deaths among adults aged 25-64 years by total ACM deaths across all ages and multiplying the result by 100. The pooled premature CMR was calculated by dividing the total number of deaths among adults aged 25-64 years by the sum of the annual population denominators for adults in the same age group over the study period and multiplying by a million. The 95% CIs for pooled mortality rates were calculated assuming a Poisson distribution of deaths, using the normal approximation given the large number of observed deaths. Calculations were performed using Microsoft Excel (Microsoft Corporation, Redmond, WA, USA).

AAMRs were obtained using direct standardization to the 2000 US standard population, the standard used by CDC WONDER for these estimates. Minor differences in the 95% confidence limits of a few AAMR estimates were observed between the 1999-2020 and 2018-2024 datasets for the overlapping years 2018-2020. Where such differences occurred, values from the 1999-2020 dataset were retained for consistency.

Annual changes in mortality counts, CMR, AAMR, and premature mortality proportion were assessed descriptively across the entire study period. For descriptive comparison, the pre-pandemic period was defined as 1999-2019, the pandemic-era period as 2020-2021, and the post-pandemic period as 2022-2024 [8]. As supplementary pooled comparisons, crude rate ratios (RRs) and 95% CIs were calculated for male versus female adults and adults aged 45-64 versus 25-44 years using the pooled death counts and person-time denominators; log-rate-ratio standard errors were estimated from the inverse death counts. Figures were generated in Microsoft Word using the Microsoft Excel plugin.

## Results

Between 1999 and 2024, ACM accounted for 13,741 deaths against a total population of 8,086,563,832, giving a pooled CMR of 1.70 per 1,000,000 population (95% CI: 1.67-1.73). Adults aged 25-64 years accounted for 9,705 of these deaths, drawn from an adult population of 4,236,198,600 in this age band, with a pooled premature CMR of 2.3 per 1,000,000 (95% CI: 2.2-2.3). Premature deaths made up 70.63% of all ACM mortality across the study period (Table 1).

**Table 1:** Annual deaths and mortality rates due to alcoholic cardiomyopathy in the United States, 1999-2024 *Minor discrepancies in 95% CI limits for AAMR estimates were observed between the 1999-2020 and 2018-2024 datasets for the overlapping years 2018-2020. In such instances, values from the 1999-2020 dataset were retained for consistency. CMR: crude mortality rate; AAMR: age-adjusted mortality rate

Year	Total deaths	Total population	Total CMR per 1,000,000 (95% CI)	AAMR per 1,000,000 (95% CI)	Premature deaths aged 25-64 years	Adults aged 25-64 years	Premature CMR per 1,000,000 (95% CI)	Premature AAMR per 1,000,000 (95% CI)	Premature mortality proportion (%)
1999	567	279,040,168	2.0 (1.9-2.2)	2.1 (1.9-2.2)	404	145,610,928	2.8 (2.5-3.0)	2.8 (2.6-3.1)	71.25
2000	486	281,421,906	1.7 (1.6-1.9)	1.7 (1.6-1.9)	327	146,992,887	2.2 (2.0-2.5)	2.3 (2.0-2.5)	67.28
2001	499	284,968,955	1.8 (1.6-1.9)	1.8 (1.6-1.9)	354	149,014,837	2.4 (2.1-2.6)	2.4 (2.1-2.6)	70.94
2002	454	287,625,193	1.6 (1.4-1.7)	1.6 (1.4-1.7)	320	150,685,821	2.1 (1.9-2.4)	2.1 (1.9-2.3)	70.48
2003	467	290,107,933	1.6 (1.5-1.8)	1.6 (1.4-1.7)	332	152,226,900	2.2 (1.9-2.4)	2.1 (1.9-2.3)	71.09
2004	412	292,805,298	1.4 (1.3-1.5)	1.4 (1.2-1.5)	285	154,002,065	1.9 (1.6-2.1)	1.8 (1.6-2.0)	69.17
2005	414	295,516,599	1.4 (1.3-1.5)	1.3 (1.2-1.5)	310	155,901,586	2.0 (1.8-2.2)	1.9 (1.6-2.1)	74.88
2006	423	298,379,912	1.4 (1.3-1.6)	1.4 (1.2-1.5)	320	157,855,252	2.0 (1.8-2.2)	1.9 (1.7-2.1)	75.65
2007	562	301,231,207	1.9 (1.7-2.0)	1.8 (1.6-1.9)	396	159,578,066	2.5 (2.2-2.7)	2.3 (2.1-2.6)	70.46
2008	551	304,093,966	1.8 (1.7-2.0)	1.7 (1.5-1.8)	394	161,017,469	2.4 (2.2-2.7)	2.2 (2.0-2.5)	71.51
2009	536	306,771,529	1.7 (1.6-1.9)	1.6 (1.5-1.7)	383	162,483,841	2.4 (2.1-2.6)	2.1 (1.9-2.4)	71.46
2010	509	308,745,538	1.6 (1.5-1.8)	1.5 (1.4-1.6)	374	163,623,999	2.3 (2.1-2.5)	2.1 (1.9-2.3)	73.48
2011	464	311,591,917	1.5 (1.4-1.6)	1.3 (1.2-1.4)	337	165,198,795	2.0 (1.8-2.3)	1.8 (1.6-2.0)	72.63
2012	509	313,914,040	1.6 (1.5-1.8)	1.5 (1.3-1.6)	380	165,680,681	2.3 (2.1-2.5)	2.1 (1.9-2.3)	74.66
2013	511	316,128,839	1.6 (1.5-1.8)	1.4 (1.3-1.5)	367	166,381,240	2.2 (2.0-2.4)	1.9 (1.7-2.1)	71.82
2014	516	318,857,056	1.6 (1.5-1.8)	1.4 (1.3-1.5)	366	167,566,069	2.2 (2.0-2.4)	1.9 (1.7-2.1)	70.93
2015	552	321,418,820	1.7 (1.6-1.9)	1.5 (1.3-1.6)	383	168,792,965	2.3 (2.0-2.5)	2.0 (1.8-2.2)	69.38
2016	537	323,127,513	1.7 (1.5-1.8)	1.4 (1.3-1.6)	387	169,397,222	2.3 (2.1-2.5)	2.1 (1.8-2.3)	72.07
2017	551	325,719,178	1.7 (1.6-1.8)	1.5 (1.3-1.6)	369	170,588,652	2.2 (1.9-2.4)	2.0 (1.8-2.2)	66.97
2018	574	327,167,434	1.8 (1.6-1.9)	1.5 (1.4-1.6)	404	170,879,997	2.4 (2.1-2.6)	2.1 (1.9-2.3)*	70.38
2019	580	328,239,523	1.8 (1.6-1.9)	1.5 (1.4-1.6)	408	170,922,904	2.4 (2.2-2.6)	2.1 (1.9-2.3)	70.34
2020	654	329,484,123	2.0 (1.8-2.1)	1.7 (1.6-1.9)	460	170,975,648	2.7 (2.4-2.9)	2.5 (2.3-2.7)*	70.34
2021	676	331,893,745	2.0 (1.9-2.2)	1.8 (1.6-1.9)	484	172,390,459	2.8 (2.6-3.1)	2.6 (2.3-2.8)	71.6
2022	635	333,287,557	1.9 (1.8-2.1)	1.6 (1.5-1.8)	445	171,713,747	2.6 (2.4-2.8)	2.4 (2.2-2.7)	70.08
2023	586	334,914,895	1.7 (1.6-1.9)	1.5 (1.4-1.6)	391	172,281,401	2.3 (2.0-2.5)	2.1 (1.9-2.3)	66.72
2024	516	340,110,988	1.5 (1.4-1.6)	1.3 (1.2-1.4)	325	174,435,169	1.9 (1.7-2.1)	1.7 (1.5-1.9)	62.98
Total/pooled	13,741	8,086,563,832	1.70 (1.67-1.73)	-	9,705	4,236,198,600	2.3 (2.2-2.3)	-	70.63

Annual deaths ranged from 412 in 2004 to 676 in 2021, and total CMR fluctuated between 1.4 per 1,000,000 in 2004-2006 and 2.0 per 1,000,000 in 1999, 2020, and 2021. Rates fell through the mid-2000s, climbed through the 2010s, peaked in 2020 and 2021, then declined to 516 deaths and a CMR of 1.5 (95% CI: 1.4-1.6) by 2024. AAMR tracked closely with CMR through most of the series but ran below it in the final years, falling from 1.7 (95% CI: 1.6-1.9) per million in 2020 to 1.3 (95% CI: 1.2-1.4) in 2024. Premature AAMR followed a similar pattern, declining from 2.5 (95% CI: 2.3-2.7) in 2020 to 1.7 (95% CI: 1.5-1.9) in 2024, against a premature CMR of 1.9 per million that year. The premature mortality proportion varied from 75.65% in 2006 to 62.98% in 2024 (Table 1, Figure 1).

**Figure 1:**
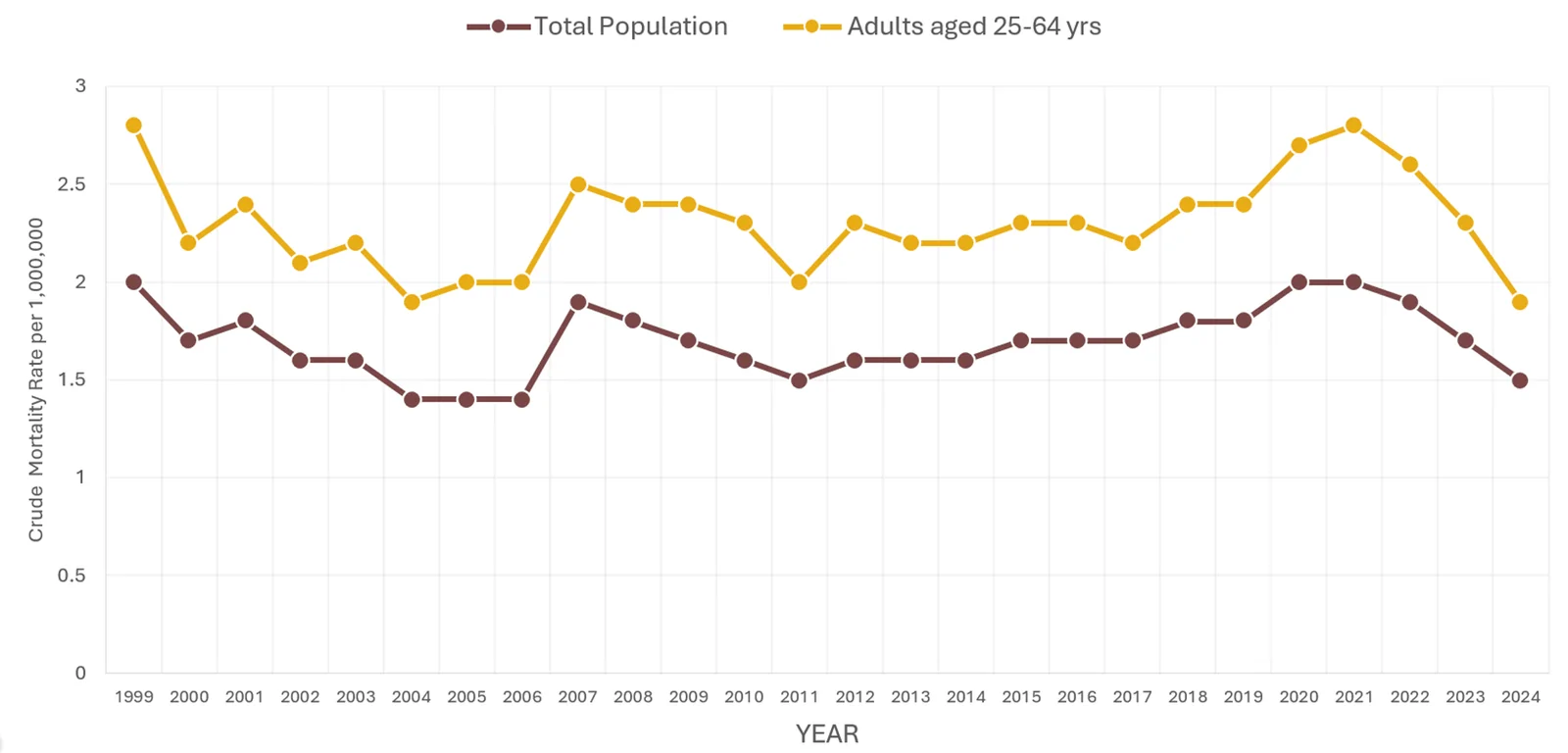
Yearly trends in crude mortality rates per 1,000,000 due to alcoholic cardiomyopathy, 1999-2024

Males accounted for 8,273 (85.24%) of the 9,705 premature deaths and females for 1,432 (14.76%). The pooled premature CMR was 3.9 per 1,000,000 (95% CI: 3.9-4.0) in males compared with 0.7 per 1,000,000 (95% CI: 0.6-0.7) in females (Table 2).

**Table 2:** Premature mortality due to alcoholic cardiomyopathy by sex and ethnicity (Hispanic origin), 1999-2024 *Ethnicity-specific proportions were calculated among records with any Hispanic origin stated (Hispanic or Latino, or non-Hispanic or Latino); records with origin not stated were excluded due to suppressed counts or unreliable data in the extracted dataset

	Subgroup	Premature deaths among adults aged 25-64 years	Adult population aged 25-64 years	Crude mortality rate per 1,000,000 (95% CI)	Proportion of premature deaths (%)
Sex	Male	8,273	2,099,484,137	3.9 (3.9-4.0)	85.24
	Female	1,432	2,136,714,463	0.7 (0.6-0.7)	14.76
Ethnicity* (Hispanic origin)	Hispanic or Latino	791	655,507,580	1.2 (1.1-1.3)	8.19*
	Non-Hispanic or Latino	8,868	3,580,691,020	2.5 (2.4-2.5)	91.81*

Using the underlying pooled counts and denominators, the male-to-female crude RR was 5.88 (95% CI: 5.56-6.22), demonstrating a marked pooled sex disparity. Annually, male CMR fell from 5.0 (95% CI: 4.5-5.5) in 1999 to a low of 3.0 (95% CI: 2.7-3.4) in 2024, with an intervening rise to 4.7 (95% CI: 4.3-5.2) in 2021; male AAMR followed a similar path but was slightly lower in recent years, at 2.8 (95% CI: 2.4-3.1) against a CMR of 3.0 in 2024. Female CMR demonstrated minor fluctuations across the period, from around 0.5 in the early 2000s to a peak of 0.9 (95% CI: 0.7-1.1) between 2020 and 2022, before easing to 0.7 in the final two years, with AAMR remaining close to CMR throughout (Table 3, Figure 2).

**Table 3:** Annual CMRs and AAMRs with 95% CIs due to alcoholic cardiomyopathy among adults aged 25-64 years by sex, 1999-2024 *Minor discrepancies in 95% CI limits for AAMR estimates were observed between the 1999-2020 and 2018-2024 datasets for the overlapping years 2018-2020. In such instances, values from the 1999-2020 dataset were retained for consistency. CMR: crude mortality rate; AAMR: age-adjusted mortality rate

Year	Sex			
	Male		Female	
	CMR per 1,000,000 (95% CI)	AAMR per 1,000,000 (95% CI)	CMR per 1,000,000 (95% CI)	AAMR per 1,000,000 (95% CI)
1999	5.0 (4.5-5.5)	5.1 (4.6-5.7)	0.6 (0.4-0.8)	0.6 (0.5-0.8)
2000	4.0 (3.5-4.4)	4.1 (3.6-4.5)	0.5 (0.4-0.7)	0.5 (0.4-0.7)
2001	4.3 (3.8-4.7)	4.3 (3.8-4.8)	0.5 (0.4-0.7)	0.5 (0.4-0.7)
2002	3.8 (3.3-4.2)	3.8 (3.3-4.2)	0.5 (0.4-0.7)	0.5 (0.4-0.7)
2003	4.0 (3.5-4.4)	3.9 (3.5-4.3)	0.4 (0.3-0.6)	0.4 (0.3-0.6)
2004	3.2 (2.8-3.6)	3.1 (2.7-3.5)	0.5 (0.4-0.7)	0.5 (0.3-0.7)
2005	3.4 (3.0-3.8)	3.2 (2.8-3.6)	0.6 (0.4-0.8)	0.6 (0.4-0.8)
2006	3.5 (3.1-3.9)	3.3 (2.9-3.7)	0.6 (0.4-0.8)	0.5 (0.4-0.7)
2007	4.4 (3.9-4.8)	4.1 (3.7-4.6)	0.6 (0.5-0.8)	0.6 (0.4-0.8)
2008	4.3 (3.9-4.8)	4.0 (3.6-4.4)	0.6 (0.5-0.8)	0.6 (0.4-0.7)
2009	4.1 (3.6-4.5)	3.8 (3.4-4.2)	0.7 (0.5-0.9)	0.6 (0.5-0.8)
2010	3.8 (3.4-4.3)	3.5 (3.1-3.8)	0.8 (0.6-1.0)	0.7 (0.5-0.9)
2011	3.5 (3.1-3.9)	3.2 (2.8-3.5)	0.6 (0.4-0.8)	0.5 (0.4-0.7)
2012	4.0 (3.6-4.4)	3.6 (3.2-4.0)	0.6 (0.5-0.8)	0.5 (0.4-0.7)
2013	3.8 (3.3-4.2)	3.3 (3.0-3.7)	0.7 (0.5-0.9)	0.6 (0.4-0.8)
2014	3.7 (3.3-4.1)	3.2 (2.9-3.6)	0.7 (0.5-0.9)	0.6 (0.5-0.8)
2015	3.8 (3.4-4.2)	3.4 (3.0-3.8)	0.8 (0.6-1.0)	0.6 (0.5-0.8)
2016	3.9 (3.5-4.4)	3.6 (3.2-4.0)	0.7 (0.5-0.9)	0.6 (0.4-0.8)
2017	3.7 (3.3-4.1)	3.4 (3.0-3.8)	0.7 (0.5-0.9)	0.6 (0.5-0.8)
2018	4.0 (3.6-4.4)	3.6 (3.2-4.0)	0.8 (0.6-1.0)	0.7 (0.6-0.9)
2019	4.1 (3.6-4.5)	3.7 (3.3-4.1)	0.7 (0.6-0.9)	0.7 (0.5-0.9)
2020	4.5 (4.1-5.0)	4.2 (3.8-4.6)*	0.9 (0.7-1.1)	0.9 (0.7-1.1)
2021	4.7 (4.3-5.2)	4.3 (3.9-4.8)	0.9 (0.7-1.1)	0.8 (0.7-1.1)
2022	4.3 (3.8-4.7)	3.9 (3.5-4.4)	0.9 (0.7-1.1)	0.9 (0.7-1.1)
2023	3.8 (3.4-4.2)	3.5 (3.2-4.0)	0.7 (0.6-0.9)	0.7 (0.5-0.9)
2024	3.0 (2.7-3.4)	2.8 (2.4-3.1)	0.7 (0.5-0.9)	0.7 (0.5-0.9)

**Figure 2:**
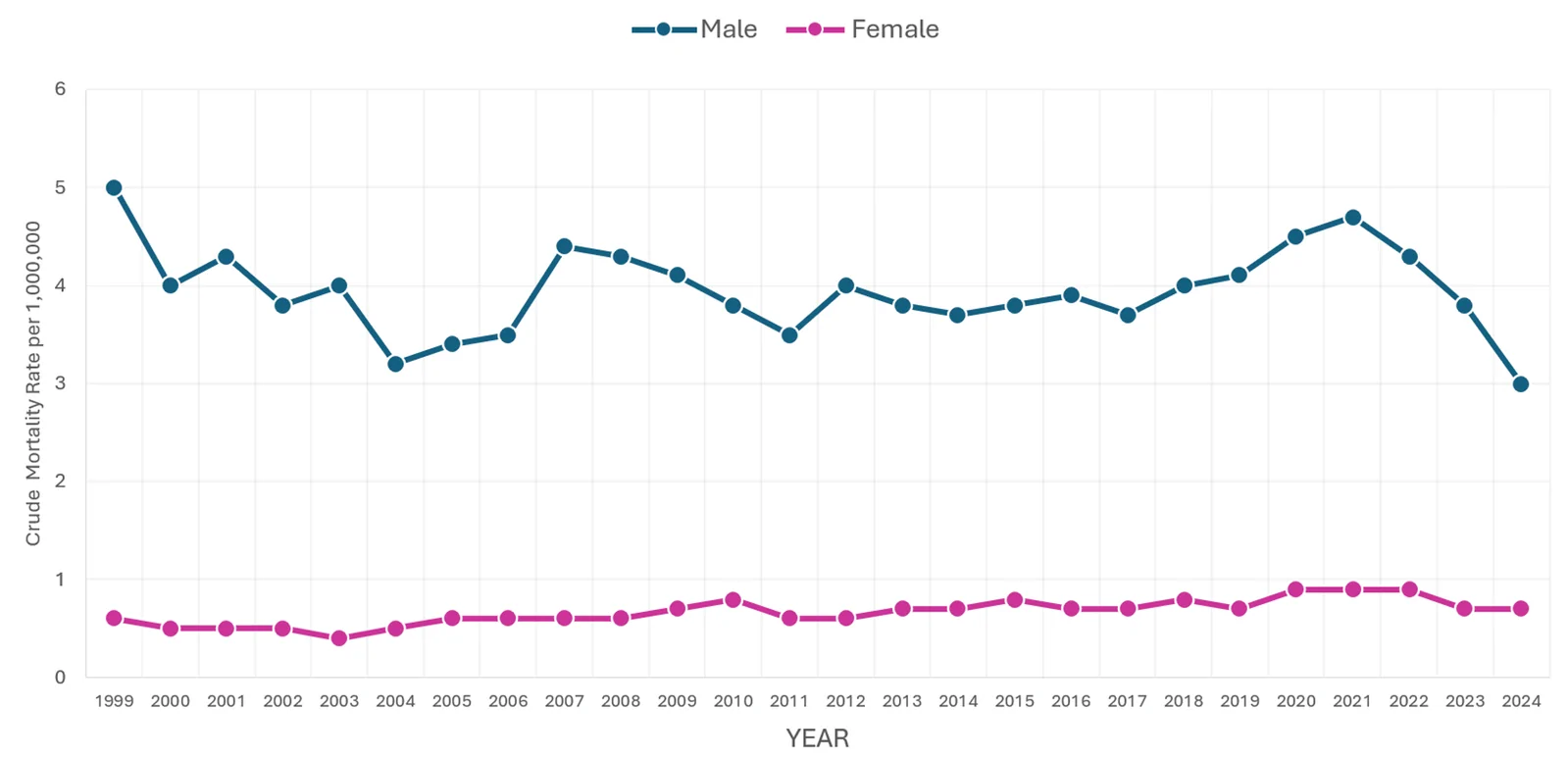
Yearly trends in crude mortality rates per 1,000,000 due to alcoholic cardiomyopathy among adults aged 25-64 years by sex, 1999-2024

Among premature deaths with stated Hispanic origin, non-Hispanic or Latino adults accounted for 8,868 (91.81%) and Hispanic or Latino adults for 791 (8.19%), with pooled CMRs of 2.5 (95% CI: 2.4-2.5) and 1.2 (95% CI: 1.1-1.3) per 1,000,000, respectively (Table 2). Annual CMR among Hispanic or Latino adults declined from 2.4 (95% CI: 1.7-3.3) in 1999 to a low of 0.9 in several years, including 2024 (95% CI: 0.6-1.2); the 2000 estimate was classed as unreliable owing to low event counts. AAMR ran notably above CMR for this group in the earlier study years, at 3.1 (95% CI: 2.2-4.3) against a CMR of 2.4 in 1999, with the gap narrowing to near parity by the mid-2010s. Non-Hispanic or Latino CMR remained within a narrower band through the 2000s and 2010s before climbing to a peak of 3.1 (95% CI: 2.8-3.4) in 2021 and falling to 2.1 (95% CI: 1.9-2.3) by 2024, with AAMR tracking just below CMR by the end of the series, at 1.9 (95% CI: 1.7-2.2) in 2024 (Table 4, Figure 3).

**Table 4:** Annual CMRs and AAMRs with 95% CIs due to alcoholic cardiomyopathy among adults aged 25-64 years by ethnicity (Hispanic origin), 1999-2024 *Ethnicity-specific proportions were calculated among records with any Hispanic origin stated (Hispanic or Latino, or non-Hispanic or Latino); records with origin not stated were excluded due to suppressed counts or unreliable data in the extracted dataset. **Rates were considered unreliable when based on low event counts and/or when the estimate did not meet CDC WONDER reliability standards because of statistical imprecision. CMR: crude mortality rate; AAMR: age-adjusted mortality rate; CDC WONDER: Centers for Disease Control and Prevention Wide-ranging Online Data for Epidemiologic Research

Year	Ethnicity*			
	Hispanic or Latino		Non-Hispanic or Latino	
	CMR per 1,000,000 (95% CI)	AAMR per 1,000,000 (95% CI)	CMR per 1,000,000 (95% CI)	AAMR per 1,000,000 (95% CI)
1999	2.4 (1.7-3.3)	3.1 (2.2-4.3)	2.8 (2.5-3.1)	2.8 (2.5-3.1)
2000	Unreliable**	Unreliable**	2.4 (2.1-2.6)	2.3 (2.1-2.6)
2001	1.4 (0.9-2.1)	1.8 (1.2-2.7)	2.5 (2.2-2.8)	2.4 (2.1-2.7)
2002	1.6 (1.1-2.3)	2.0 (1.3-2.9)	2.2 (1.9-2.4)	2.1 (1.8-2.3)
2003	1.4 (0.9-2.0)	1.7 (1.1-2.6)	2.3 (2.0-2.5)	2.1 (1.9-2.4)
2004	1.2 (0.8-1.8)	1.6 (1.0-2.3)	1.9 (1.7-2.2)	1.8 (1.6-2.0)
2005	1.1 (0.7-1.7)	1.3 (0.8-1.9)	2.1 (1.9-2.3)	1.9 (1.7-2.1)
2006	1.5 (1.0-2.1)	1.7 (1.2-2.4)	2.1 (1.9-2.4)	1.9 (1.7-2.1)
2007	1.5 (1.0-2.1)	1.7 (1.2-2.4)	2.6 (2.4-2.9)	2.4 (2.1-2.6)
2008	1.2 (0.8-1.8)	1.4 (1.0-2.1)	2.6 (2.4-2.9)	2.3 (2.1-2.6)
2009	1.1 (0.7-1.6)	1.4 (0.9-2.0)	2.6 (2.3-2.8)	2.3 (2.0-2.5)
2010	0.9 (0.6-1.4)	1.0 (0.6-1.6)	2.5 (2.3-2.8)	2.2 (2.0-2.4)
2011	1.1 (0.7-1.6)	1.2 (0.8-1.8)	2.2 (1.9-2.4)	1.9 (1.7-2.1)
2012	0.9 (0.5-1.3)	0.9 (0.6-1.4)	2.6 (2.3-2.8)	2.2 (2.0-2.5)
2013	1.2 (0.8-1.7)	1.3 (0.9-1.9)	2.4 (2.1-2.7)	2.0 (1.8-2.3)
2014	1.2 (0.8-1.7)	1.3 (0.9-1.8)	2.4 (2.1-2.6)	2.0 (1.8-2.2)
2015	1.0 (0.6-1.4)	1.0 (0.7-1.5)	2.5 (2.3-2.8)	2.1 (1.9-2.4)
2016	1.2 (0.8-1.6)	1.2 (0.8-1.7)	2.5 (2.2-2.8)	2.2 (2.0-2.4)
2017	1.0 (0.6-1.4)	1.0 (0.6-1.4)	2.4 (2.1-2.7)	2.2 (1.9-2.4)
2018	0.9 (0.6-1.4)	1.0 (0.6-1.4)	2.7 (2.4-2.9)	2.3 (2.1-2.6)
2019	1.4 (1.1-1.9)	1.5 (1.1-2.0)	2.6 (2.3-2.8)	2.3 (2.0-2.5)
2020	1.3 (0.9-1.7)	1.3 (1.0-1.8)	3.0 (2.7-3.3)	2.7 (2.4-3.0)
2021	1.5 (1.1-2.0)	1.6 (1.2-2.1)	3.1 (2.8-3.4)	2.8 (2.5-3.0)
2022	1.2 (0.8-1.6)	1.2 (0.8-1.6)	2.9 (2.6-3.2)	2.6 (2.4-2.9)
2023	1.1 (0.7-1.5)	1.1 (0.8-1.5)	2.5 (2.3-2.8)	2.3 (2.0-2.6)
2024	0.9 (0.6-1.2)	0.9 (0.6-1.2)	2.1 (1.9-2.3)	1.9 (1.7-2.2)

**Figure 3:**
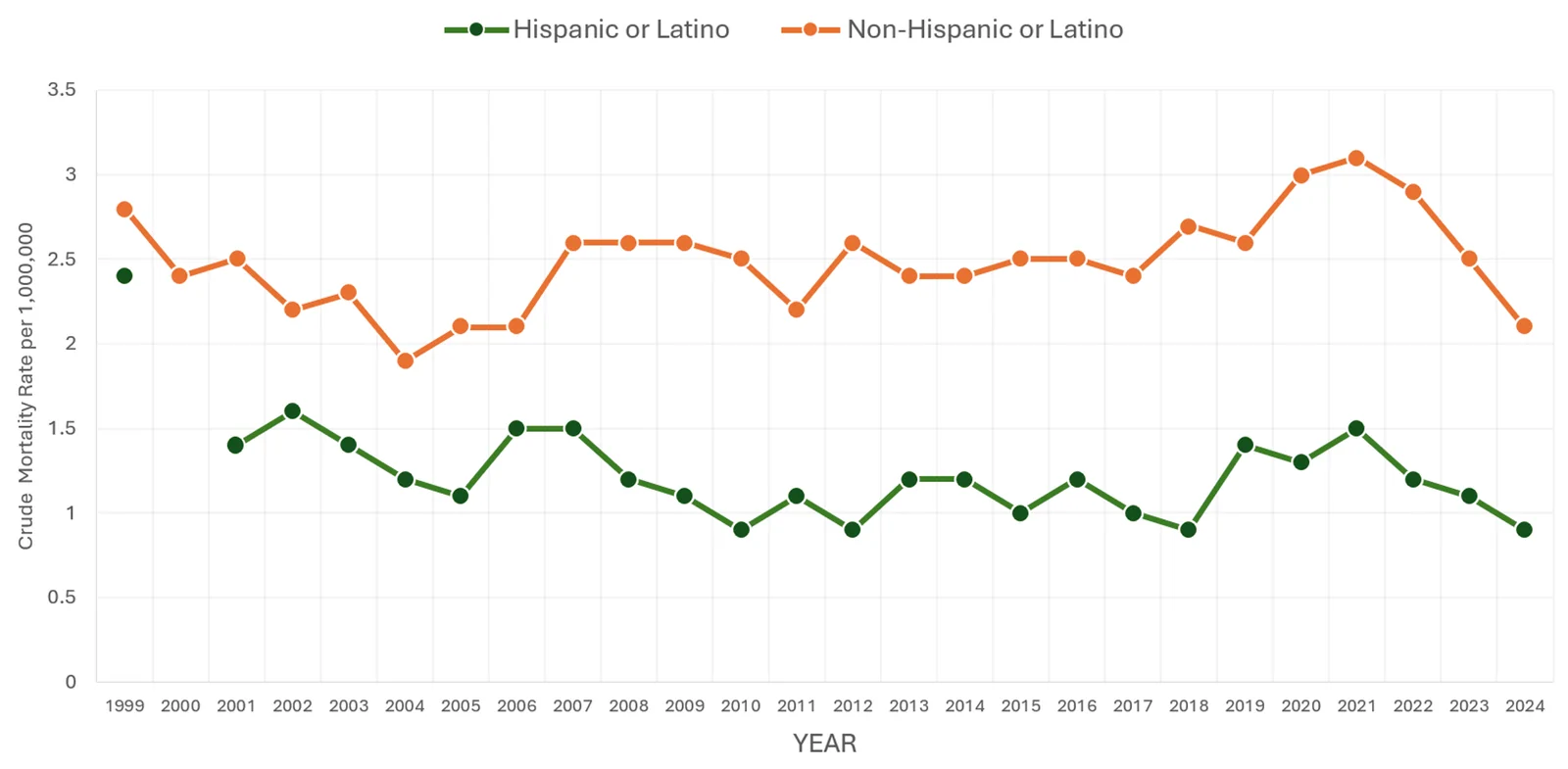
Yearly trends in crude mortality rates per 1,000,000 due to alcoholic cardiomyopathy among adults aged 25-64 years by ethnicity (Hispanic or Latino origin), 1999-2024

Adults aged 45-64 years accounted for 7,647 (78.79%) of premature deaths and those aged 25-44 years for 2,058 (21.21%), with pooled CMRs of 3.8 (95% CI: 3.7-3.9) and 0.9 (95% CI: 0.9-1.0) per 1,000,000, respectively (Table 5).

**Table 5:** Premature mortality due to alcoholic cardiomyopathy by age subgroup, 1999-2024

Age subgroup	Premature deaths in respective age subgroup	Adult population in respective age subgroup	Crude mortality rate per 1,000,000 (95% CI)	Proportion of premature deaths (%)
25-44 years	2,058	2,211,398,678	0.9 (0.9-1.0)	21.21
45-64 years	7,647	2,024,799,922	3.8 (3.7-3.9)	78.79

The pooled crude RR for adults aged 45-64 versus 25-44 years was 4.06 (95% CI: 3.87-4.26). CMR in the 45-64 group fell from 4.8 (95% CI: 4.3-5.4) in 1999 to a trough of 3.2 to 3.4 between 2004 and 2006, rose to a secondary peak of 4.3 (95% CI: 3.9-4.8) in 2021, then dropped to 2.8 (95% CI: 2.4-3.1) in 2024, its lowest point across the series; AAMR in this group eased slightly below CMR by 2024, at 2.5 (95% CI: 2.2-2.9). The 25-44 group showed a similar but smaller shift, falling from 1.3 in 1999 to 0.6 between 2013 and 2014, climbing to 1.4 in 2021 and 2022, and settling at 1.0 (95% CI: 0.8-1.3) in 2024, with AAMR closely matching CMR throughout this subgroup (Table 6, Figure 4).

**Table 6:** Annual CMRs and AAMRs with 95% CIs due to alcoholic cardiomyopathy among adults aged 25-64 years by age subgroup, 1999-2024 *Minor discrepancies in 95% CI limits for AAMR estimates were observed between the 1999-2020 and 2018-2024 datasets for the overlapping years 2018-2020. In such instances, values from the 1999-2020 dataset were retained for consistency. CMR: crude mortality rate; AAMR: age-adjusted mortality rate

Year	Age subgroup			
	Age 25-44 years		Age 45-64 years	
	CMR per 1,000,000 (95% CI)	AAMR per 1,000,000 (95% CI)	CMR per 1,000,000 (95% CI)	AAMR per 1,000,000 (95% CI)
1999	1.3 (1.1-1.6)	1.4 (1.1-1.6)	4.8 (4.3-5.4)	4.8 (4.3-5.4)
2000	1.0 (0.8-1.2)	1.0 (0.8-1.2)	3.9 (3.4-4.4)	3.9 (3.4-4.4)
2001	1.0 (0.8-1.3)	1.1 (0.9-1.3)	4.1 (3.6-4.6)	4.2 (3.7-4.7)
2002	1.0 (0.8-1.2)	1.0 (0.8-1.2)	3.6 (3.1-4.1)	3.6 (3.1-4.0)
2003	0.8 (0.6-1.0)	0.8 (0.6-1.0)	3.9 (3.4-4.3)	3.9 (3.4-4.4)
2004	0.7 (0.5-0.9)	0.7 (0.6-1.0)	3.2 (2.8-3.6)	3.2 (2.8-3.6)
2005	0.7 (0.5-0.9)	0.7 (0.6-0.9)	3.4 (3.0-3.9)	3.4 (2.9-3.8)
2006	0.9 (0.7-1.1)	0.9 (0.7-1.2)	3.3 (2.9-3.7)	3.3 (2.8-3.7)
2007	1.0 (0.8- 1.2)	1.1 (0.8-1.3)	4.1 (3.6-4.5)	4.0 (3.6-4.5)
2008	0.8 (0.6-1.0)	0.8 (0.7-1.1)	4.1 (3.7-4.6)	4.1 (3.6-4.5)
2009	0.8 (0.7-1.1)	0.8 (0.7-1.1)	3.9 (3.5-4.3)	3.9 (3.5-4.3)
2010	0.8 (0.6-1.0)	0.8 (0.6-1.1)	3.8 (3.4-4.2)	3.7 (3.3-4.1)
2011	0.7 (0.6-0.9)	0.7(0.5-0.9)	3.3 (3.0-3.7)	3.3 (2.9-3.6)
2012	0.7 (0.5-0.9)	0.8 (0.6-1.0)	3.9 (3.5-4.3)	3.9 (3.4-4.3)
2013	0.6 (0.5-0.8)	0.6 (0.5-0.8)	3.8 (3.4-4.2)	3.7 (3.3-4.1)
2014	0.6 (0.5-0.8)	0.6 (0.5-0.8)	3.8 (3.3-4.2)	3.6 (3.2-4.0)
2015	0.7 (0.5-0.9)	0.7 (0.5-0.9)	3.9 (3.5-4.3)	3.7 (3.3-4.1)
2016	0.9 (0.7-1.1)	0.9 (0.7-1.2)	3.7 (3.3-4.1)	3.6 (3.2-4.0)
2017	0.9 (0.7-1.1)	0.9 (0.7-1.2)	3.5 (3.1-3.9)	3.4 (3.0-3.8)
2018	0.9 (0.7-1.1)	1.0 (0.8-1.2)	3.9 (3.4-4.3)	3.7 (3.3-4.1)
2019	1.0 (0.8-1.2)	1.0 (0.8-1.2)	3.9 (3.5-4.3)	3.6 (3.2-4.0)*
2020	1.3 (1.1-1.5)	1.4 (1.2-1.7)	4.2 (3.7-4.6)	3.9 (3.5-4.4)
2021	1.4 (1.1-1.6)	1.4 (1.2-1.7)	4.3 (3.9-4.8)	4.1 (3.7-4.6)
2022	1.4 (1.2-1.6)	1.5 (1.3-1.8)	3.9 (3.5-4.3)	3.6 (3.2-4.1)
2023	1.1 (0.9-1.3)	1.2 (0.9-1.4)	3.5 (3.1-3.9)	3.3 (2.9-3.7)
2024	1.0 (0.8-1.3)	1.1 (0.9-1.3)	2.8 (2.4-3.1)	2.5 (2.2-2.9)

**Figure 4:**
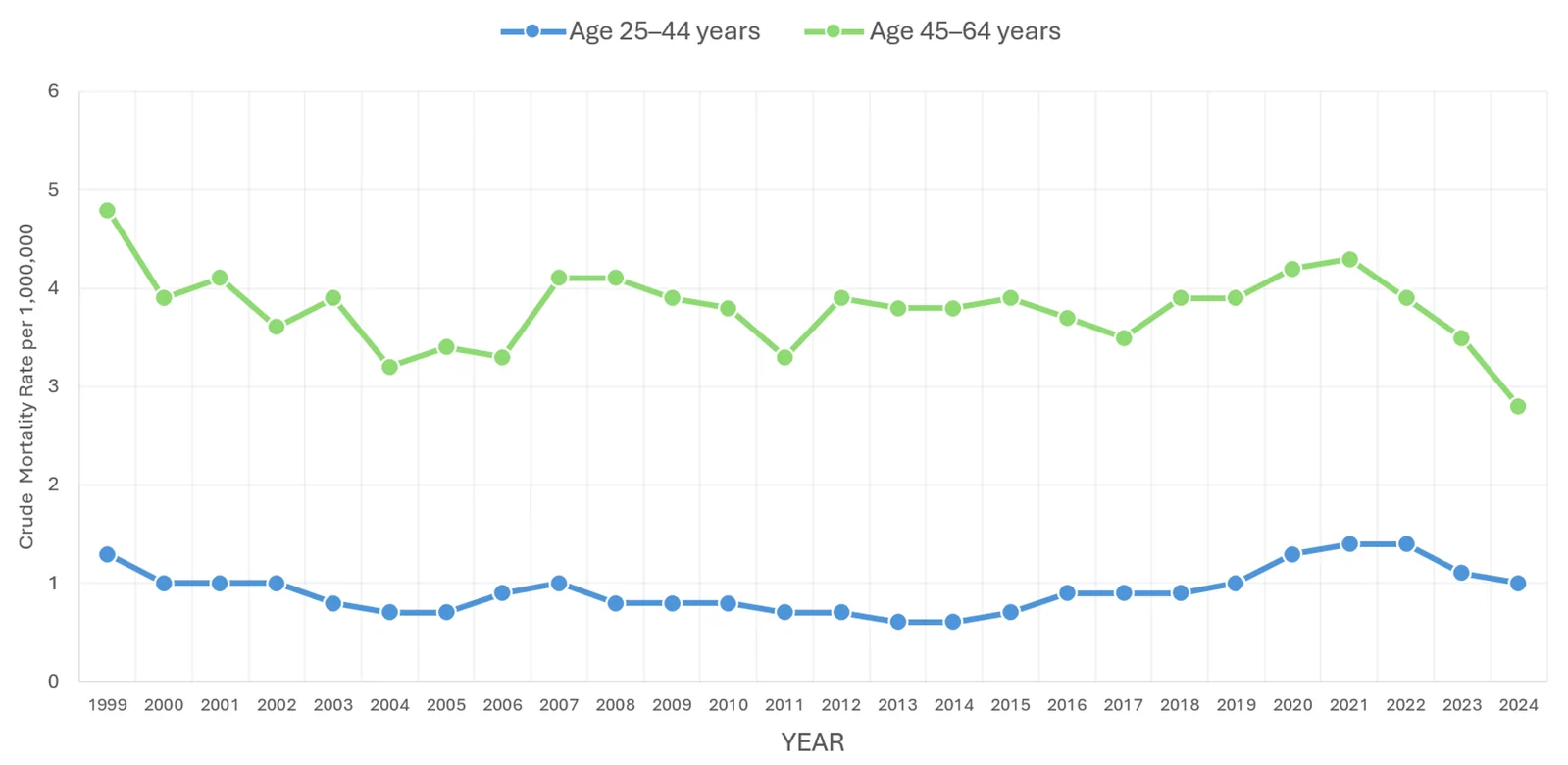
Yearly trends in crude mortality rates per 1,000,000 due to alcoholic cardiomyopathy among adults aged 25-64 years by age subgroup, 1999-2024

Looking at the data over the specified pandemic periods, there was a clear change over time. In the pre-pandemic period, mortality fell throughout the mid-2000s but then increased gradually in the 2010s. This rise in mortality became more noticeable during the pandemic period (2020-2021), when the overall CMR reached 2.0 per 1,000,000 in both 2020 (95% CI: 1.8-2.1) and 2021 (95% CI: 1.9-2.2), and the number of annual deaths peaked at 676 in 2021. In the post-pandemic period (2022-2024), mortality decreased step by step, ending in 2024 with 516 deaths and a CMR of 1.5 (95% CI: 1.4-1.6) per 1,000,000. Premature mortality also followed the same pattern, the CMR falling from 2.8 (95% CI: 2.6-3.1) per 1,000,000 in 2021 to 1.9 (95% CI: 1.7-2.1) per 1,000,000 in 2024, and the share of deaths among adults aged 25-64 years dropping from 71.60% to 62.98% by 2024.

## Discussion

In this nationwide analysis of the US mortality data spanning 1999-2024, ACM accounted for 13,741 deaths, of which 9,705 (70.6%) occurred among adults aged 25-64 years. The concentration of ACM mortality within this working-age population is an important finding because death from a potentially preventable alcohol-related cardiovascular condition during economically and socially productive years represents a burden that extends beyond cardiovascular mortality alone. Premature mortality was strongly concentrated among men and adults aged 45-64 years. Mortality increased during 2020-2021, with the highest observed burden in 2021, followed by a progressive decline through 2024. Collectively, these findings reveal that the epidemiology of ACM mortality has not been static over the past quarter century and that both long-term secular changes and more recent population-level disruptions may have influenced its trajectory. However, since this is an ecological descriptive analysis, these temporal observations are interpreted as population-level patterns rather than evidence of causation or formally estimated change points.

Temporal pattern of ACM mortality

The overall temporal pattern in this study is characterized by an initial decline in mortality during the early 2000s, followed by a gradual increase during the subsequent decade, a marked elevation during 2020-2021, and a decline thereafter. The pre-pandemic trajectory is broadly consistent with previous US analyses, although comparisons require consideration of differences in study design, age definitions, and data sources. Zuin et al. reported increasing ACM-attributed mortality in the US between 2010 and 2020, particularly among men and younger-to-middle-aged adults [9]. In contrast, the analysis by Daise et al., based on Global Burden of Disease data through 2019, reported an overall decline in ACM mortality over the longer pre-pandemic period [10]. These apparently different observations are not necessarily contradictory. They may reflect differences in the population examined, the underlying mortality metric, coding and estimation methodology, and the specific time intervals evaluated. The present analysis extends mortality surveillance through 2024 and therefore provides an opportunity to place the pandemic-era increase within a longer temporal context.

The increase in ACM mortality observed in 2020 and 2021 is particularly noteworthy. Mortality from ACM increased concurrently with major disruptions in healthcare delivery, social structures, employment, and alcohol-use patterns during the COVID-19 pandemic [11]. Previous work has demonstrated substantial changes in cardiovascular mortality during and after the pandemic, with important temporal and sociodemographic disparities [8]. However, the present ecological mortality analysis cannot establish that the pandemic itself caused the increase in ACM mortality. Several mechanisms may have contributed, including changes in alcohol consumption, delayed or reduced access to healthcare, interruption of addiction-treatment services, worsening psychosocial stressors, and altered patterns of presentation and diagnosis [12-15]. Conversely, the decline in mortality after 2021 should not be interpreted as evidence that the underlying burden of alcohol-related cardiac disease has resolved. Changes in healthcare utilization, mortality certification, alcohol exposure, or the composition of high-risk populations could also contribute to the observed post-pandemic pattern [16,17].

Importantly, the 2024 premature ACM mortality rate remained measurable despite the decline from the pandemic-era peak. This persistence supports viewing ACM as an ongoing cardiovascular prevention challenge instead of exclusively a consequence of an acute period of social disruption.

Disproportionate burden among men

One of the most consistent findings was the marked sex disparity in premature ACM mortality. Men accounted for 85.2% of premature deaths, and the pooled CMR was approximately six times that in women. This observation is in line with the existing literature that indicates a significantly higher prevalence and burden of alcohol-related cardiomyopathy in men [10,18]. The fact that this difference does not disappear over the whole study period indicates that the sex disparity is not merely a transient phenomenon.

Several factors may contribute to this pattern. Differences in the prevalence and intensity of alcohol exposure are likely to be important, as are differences in patterns of heavy drinking and cumulative exposure. It is also possible that biological susceptibility, comorbid cardiovascular risk factors, healthcare-seeking behavior, and variations in recognition and treatment of alcohol-use disorders all play a role. However, the available mortality data through CDC WONDER cannot differentiate between these possible mechanisms [18]. The magnitude and consistency of the observed disparity nevertheless have practical implications: men with sustained heavy alcohol exposure, particularly those in middle age, may represent an important target population for cardiovascular risk assessment, alcohol-use screening, and early evaluation for alcohol-related myocardial injury.

Simultaneously, the comparatively low mortality rate of women cannot be viewed as evidence of low risk. The female mortality rate also increased during the pandemic period, reaching approximately 0.9 per million in 2020-2022. This observation is relevant because alcohol-related harm among women has received increasing attention, and population-level surveillance should avoid framing ACM as an exclusively male disease.

Age-related concentration of premature mortality

Age was another prominent determinant of ACM mortality. Nearly four-fifths (78.8%) of premature deaths occurred among adults aged 45-64 years, whose pooled CMR was more than four times that of adults aged 25-44 years. This trend is biologically plausible considering that ACM is normally a cumulative exposure and myocardial injury that develops over time. The significantly greater burden in older working adults could thus be a result of longer exposure to alcohol and the accrual of cardiovascular and systemic comorbidity [19]. Chronic ethanol exposure can produce cumulative myocardial injury, oxidative stress, myocyte dysfunction, and adverse ventricular remodeling, which may progressively increase the clinical burden of cardiomyopathy over time [20].

The age-specific temporal pattern is also informative. Both age groups demonstrated an increase around 2020-2022, but the absolute mortality burden remained considerably higher among adults aged 45-64 years. This evidence indicates that prevention strategies should not target only very young adults. However, prior detection of detrimental drinking habits and cardiovascular effects in the earlier decades of life can offer a chance to avoid the development of clinically overt cardiomyopathy in later working years.

From a public-health perspective, the concentration of deaths in adults aged 45-64 years is particularly consequential. Death during this life period can be a severe loss of productive life years and can impact household income, dependents, and the overall productivity of society. Therefore, the role of ACM in this population is not confined to its contribution to the cardiovascular mortality statistics.

Hispanic origin and the importance of social determinants

The analysis showed that the rate of mortality was higher in the non-Hispanic or Latino group as compared to the Hispanic or Latino group. This finding requires cautious interpretation. The analysis relied on Hispanic origin as reported on death certificates and did not give a detailed evaluation of racial or ethnic subgroups, socioeconomic factors, alcohol-use behavior, or access to healthcare. In this light, the difference observed should not be construed as an indication of inherently low or high biological susceptibility on the basis of Hispanic origin.

The relationship between social context and ACM mortality is particularly relevant in this regard. According to the results of Shahid et al., the higher the social vulnerability of the county, the higher the ACM mortality of both the Hispanic and non-Hispanic population [21]. This can also be extended to imply that the outcome of ACM across population groups can be influenced by socioeconomic deprivation, access to health care, and community exposure to alcohol, social instability, and other structural variables. The reduced mortality rate in Hispanic or Latino adults observed in the current analysis could thus be due to variations in population composition, alcohol use, migration and demographic factors, access to care, diagnosis, or death-certificate classification instead of a mere ethnic effect.

While race categories could not be examined in the present analysis due to suppressed aggregate data, future studies can jointly examine race and ethnicity with socioeconomic status, geographic location, insurance coverage, healthcare access, alcohol-use behavior, and indicators of social vulnerability. Such approaches may help identify communities in which preventable ACM mortality is concentrated and in which targeted interventions could have the greatest effect.

Clinical and public health implications

The findings have several implications for cardiovascular prevention. First, ACM should be considered within the broader framework of alcohol-related cardiovascular disease rather than as an isolated manifestation of chronic alcohol exposure. Recognition of heavy alcohol use in clinical practice provides an opportunity to identify individuals before irreversible myocardial dysfunction develops. Assessment of alcohol consumption should therefore be incorporated into cardiovascular risk evaluation, particularly in patients with unexplained left ventricular dysfunction, heart failure, arrhythmias, or other clinical features compatible with alcohol-related myocardial injury.

Second, the concentration of mortality among men and adults aged 45-64 years provides potentially actionable targets for prevention. Screening for unhealthy alcohol use, appropriate counseling, referral for treatment of alcohol-use disorders, and cardiovascular evaluation in high-risk individuals may provide opportunities to interrupt disease progression. Such interventions should ideally be integrated rather than separated into cardiovascular and addiction-care pathways.

Third, the pandemic-associated increase highlights the importance of maintaining continuity of both cardiovascular and alcohol-related healthcare during periods of major healthcare-system disruption. The subsequent decline in mortality is encouraging, but continued surveillance is necessary to determine whether this represents a sustained change or a return toward the longer-term trajectory.

Finally, the substantial proportion of ACM deaths occurring before age 65 emphasizes the importance of prevention earlier in adulthood. The cardiovascular benefits of public-health measures that minimize adverse alcohol exposure can be far more extensive than the prevention of alcohol-use disorder itself. The potential effect on premature mortality makes alcohol-related cardiovascular prevention relevant to health-system planning, occupational health, and broader population-health policy.

Strengths and limitations

This study has several strengths. It uses a large, nationally representative mortality database and covers a 26-year period, allowing assessment of long-term trends as well as the pandemic and post-pandemic periods. The analysis further assesses mortality by sex, age, and Hispanic origin, presenting a more in-depth description of populations with disproportional mortality.

There are, however, several limitations that should be taken into consideration when interpreting these findings. To begin with, the CDC WONDER mortality data are based on death certificates, thus vulnerable to diagnostic misclassification, incomplete documentation, and temporal changes in diagnostic awareness, testing, and coding or certification practices. Such changes could contribute to secular trends independent of true changes in ACM incidence or case fatality, and the present data cannot quantify their contribution. ACM may also be under-recognized when multiple cardiovascular or systemic comorbidities are present; accordingly, the estimates represent mortality certified with ACM as the underlying cause rather than the population incidence of alcohol-related myocardial disease. Second, the database lacks information at the individual level about the amount, duration, or pattern of alcohol intake. It also does not allow evaluation of left ventricular performance, severity of disease, presence of comorbid coronary or valvular disease, drugs, abstinence, alcohol-use disorder treatment, or other clinical predictors of outcome. The analysis is thus unable to determine a dose-response relationship between alcohol exposure and mortality. Third, socioeconomic status, healthcare access, insurance status, geographic variation, race, and social vulnerability were not incorporated into the primary analysis. These factors may be important mediators or modifiers of ACM mortality and could partially explain the observed differences between population groups. Likewise, the classification of Hispanic origin was developed from death certificate reporting, which may be subject to misclassification relative to self-reported population data. Moreover, suppressed or unreliable figures may have affected subgroup estimates in stratified analysis by Hispanic origin. Race could not be examined as a primary stratification due to exploratory extraction revealing substantial suppression or unreliable annual estimates for some racial groups. Fourth, AAMRs were standardized to the 2000 US standard population, consistent with CDC WONDER, so absolute AAMRs may differ from studies using more recent standard populations. No alternative-standard-population sensitivity analysis was performed. Finally, this was a descriptive ecological study without formal join point or segmented trend modeling; it can characterize observed temporal patterns but cannot establish statistical change points, individual-level associations, or a causal pandemic effect.

Future directions

The results offer a rationale for more detailed studies connecting mortality surveillance with clinical, behavioral, and socioeconomic information. A feasible next step would combine individual-level alcohol-consumption information from repeated national surveys with healthcare-utilization and claims or electronic-health-record data. Geographic studies that involve social vulnerability and healthcare access can also help to pinpoint especially vulnerable populations. Interrupted time-series or difference-in-differences designs using pre-pandemic trends and suitable comparison outcomes could more directly test whether the 2020-2021 peak tracked changes in alcohol exposure, healthcare disruption, or mortality certification. Record-linkage or prospective cohort studies containing alcohol exposure, abstinence, treatment, cardiovascular phenotype, and outcomes would provide stronger individual-level evidence for decreased heart failure development and cardiovascular mortality. Validation studies of death-certificate coding could assess whether temporal changes in certification contributed to the observed secular pattern. Causal explanations for the pandemic-era pattern require individual-level or quasi-experimental studies.

## Conclusions

This study shows that ACM continues to be a significant cause of early death among adults aged 25-64 years in the US between 1999 and 2024, and that over two-thirds of all ACM deaths fall in this working-age group. Men and adults aged 45-64 years were also disproportionately represented in mortality, which was higher in 2020-2021 and fell after 2024. The fact that the mortality of ACM remains high despite the post-pandemic decrease confirms that ACM is a continuing rather than merely transient public health problem, and there is a need for long-term cardiovascular and alcohol-related prevention programs. The increased incorporation of alcohol-use screening, early cardiovascular evaluation, addiction therapy, long-term risk reduction, and continued surveillance could offer a chance to decrease preventable deaths in ACM-related conditions in the economically productive years of adulthood.
